# Sensory processing by motoneurons: a numerical model for low-level flight control in flies

**DOI:** 10.1098/rsif.2018.0408

**Published:** 2018-08-29

**Authors:** Jan Bartussek, Fritz-Olaf Lehmann

**Affiliations:** Institute of Biological Sciences, Department of Animal Physiology, University of Rostock, 18059 Rostock, Germany

**Keywords:** motor control, muscle power output, sensory integration, numerical modelling, insect flight

## Abstract

Rhythmic locomotor behaviour in animals requires exact timing of muscle activation within the locomotor cycle. In rapidly oscillating motor systems, conventional control strategies may be affected by neural delays, making these strategies inappropriate for precise timing control. In flies, wing control thus requires sensory processing within the peripheral nervous system, circumventing the central brain. The underlying mechanism, with which flies integrate graded depolarization of visual interneurons and spiking proprioceptive feedback for precise muscle activation, is under debate. Based on physiological parameters, we developed a numerical model of spike initiation in flight muscles of a blowfly. The simulated Hodgkin–Huxley neuron reproduces multiple experimental findings and explains on the cellular level how vision might control wing kinematics. Sensory processing by single motoneurons appears to be sufficient for control of muscle power during flight in flies and potentially other flying insects, reducing computational load on the central brain during body posture reflexes and manoeuvring flight.

## 1. Introduction

Rhythmic locomotor behaviour in animals results from periodic production of muscle mechanical power. Muscle power typically depends on neural activation frequency, but also strongly on the muscle's spike phase, i.e. the timing of electrical muscle activation within the locomotor cycle [1–3]. The latter mechanism provides the nervous system with an additional opportunity to influence motor control and locomotor efficacy. In most animals, the spike phase is controlled by neural feedback acting through the physiology and biomechanics of the locomotor apparatus. In the low-frequency locomotor systems of vertebrates, timing is typically achieved by cyclic output of neural central-pattern generators [4] that determine locomotor period and movements of body appendages. Conventional neural strategies for phase control, however, may fail in locomotor systems with high oscillatory frequencies of up to approximately 800 Hz [5]. Several problems for phase control in locomotor systems are associated with synaptic delays and the time needed for spike propagation from sensors to the central brain and locomotor muscles [6,7]. Thus, locomotion often requires mechanisms of sensory processing at the level of the peripheral nervous system [4]. This study investigates sensory processing and motor control in an insect that flaps its wings at approximately 150 Hz stroke frequency.

**Figure 1.**
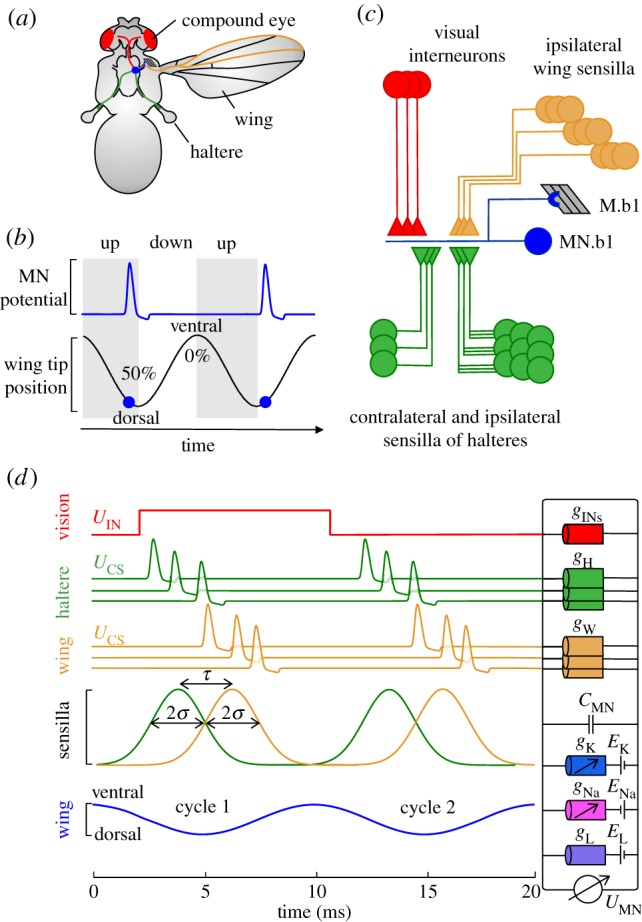
Sensory inputs to a model motoneuron. (*a*) Sensory inputs to the motoneuron (MN.b1, blue) of a basalare 1 flight control muscle (M.b1) in flies. MN.b1 receives ipsi- and contralateral input from up to 50 descending interneurons (IN) (red) and afferences of mechanoreceptive campaniform sensilla located on halteres (green) and wings (orange) [12–15]. (*b*) MN.b1 fires one spike in every wing stroke cycle at the end of the upstroke (blue). The stroke cycle begins at the ventral stroke reversal (0/100% cycle). (*c*) Conceptional pathways of MN.b1 sensory inputs. Triangles and half circles indicate electrical and chemical synapses, respectively, and circles the neurons' soma. (*d*) Hodgkin–Huxley type model of MN.b1. Visual signalling consists of graded potentials (*U*_IN_). Haltere and wing signalling (*U*_CS_) is simulated by periodic trains of action potentials with changing variance (*σ*, standard deviation), accumulating in two volleys of spikes per stroke cycle (up to 110 sensilla each, contralateral haltere is not considered). MN.b1 membrane parameters are shown on the right. *τ*, time between mean phase of sensilla activation of ipsilateral wing and haltere; *g*_INs_, total synaptic conductance between all visual IN and MN.b1; *g*_W_, conductance for one wing sensilla; *g*_H_, conductance for one haltere sensilla; *C*_MN_, membrane capacitance; *g*_K_, *g*_Na_ and *g*_L_, conductances for potassium, sodium and leak currents, respectively; *E*_K_, *E*_Na_ and *E*_L_, reversal potentials of potassium, sodium and other ions, respectively; *U*_MN_, membrane potential.

Precision requirements for muscle activation timing are pronounced during wing flapping in insects. Flies, for example, steer and manoeuvre within few 5–10 ms wing strokes [8–11]. In these animals, activation precision results from cyclic proprioceptive feedback generated by halteres and wings (figure 1*a,b*). Mechanoreceptors, i.e. campaniform sensilla, on these structures deliver timing cues, producing temporal phase-locked action potentials in every flapping cycle [8,16]. This feedback tightly couples muscle activation phase to the motor cycle, with microsecond precision in muscle spike initiation. During flight manoeuvres and body instabilities, inertial forces deflect the beating halteres from their normal stroke plane. As a result, cuticular stress causes an increase in active sensilla and alters the timing of flight muscle spike initiation [16–18]. Halteres thus act as a gyroscopic system that automatically stabilizes the fly body in flight with high accuracy and small delays [19–22].

Similar to halteres, mechanosensors on the wing surface produce wing stroke-synchronous feedback for motoneurons [8,23–25]. Their distribution and activation properties enable them to encode wing loading [26] and cyclic wing deformation [27]. Flight requires input from both sensory systems, and—although both pathways provide excitatory neural feedback—they act antipodal on wing stroke amplitude control in flies [28]. Mechanosensory neurons from halteres and wings partially circumvent the central brain and directly project onto motoneurons of wing steering muscles via enlarged chemical and electrical synapses (figure 1*c*) [23,29,30]. However, because chemical transmission rapidly fatigues at natural locomotor frequencies, proprioceptive signalling in flies is primarily transmitted via electrical synapses [29]. This signalling typically generates no more than a single steering muscle action potential per wing stroke at flapping frequencies of approximately 100–125 Hz in the blowfly *Calliphora* and approximately 200 Hz the fruit fly *Drosophila* [8,31–33].

During manoeuvring flight, the visual system of flies controls steering muscle power output by either gating muscle spiking or by shifting spike initiation phase via visual motion-sensitive descending interneurons (IN) [34–43]. These two strategies reflect two significant requirements for flight control: elevated changes of wing kinematics during saccadic flight turns and escape behaviours, and subtle changes during posture stabilization and heading precision control, respectively [10,33,44–49]. The graded changes in membrane potential of visual IN during vision-guided flight are tiny though, amounting to approximately 10 mV in *Calliphora* [38] and approximately 4 mV in *Drosophila* [12].

The first axillary (M.I1) and second basalare (M.b2) steering muscles in flies belong to the group of phasic active, visually gated flight muscles. Their activity can be switched on or off by the visual system [33,34]. On average, M.I1 and M.b2 motoneurons generate muscles spikes with approximately one-third wing stroke frequency [32]. Activation of M.I1 decreases, while activation of M.b2 increases wing stroke amplitudes during vision-guided flight turns. In addition, the gated spikes are phase-locked with respect to the stroke cycle to maximize the muscle's biomechanical efficacy [1,2,31,32]. The first basalare steering muscle (M.b1), by contrast, belongs to the group of tonic active muscles [34]. M.b1 is not gated and generates a single muscle spike in almost every wing stroke cycle in *Calliphora* and *Drosophila* [8,50–52]. Experiments have shown that M.b1 activity is crucial for maintaining elevated wing stroke amplitudes [52]. Visual stimulation of the animal's compound eyes with moving visual patterns shifts M.b1 spiking phase by up to approximately 11% of the wing stroke cycle [32,52].

M.b1 is innervated by a single motoneuron, MN.b1. Up to now, no mechanistic model exists that explains how the tiny fluctuations in membrane potential of visual IN control spike timing of MN.b1 in the presence of spiking input from mechanoreceptors. Moreover, because sensory inputs establish electrical synapses on motoneurons [12,13,25,29,30], spike phase constancy may not result from a balance between excitatory and inhibitory postsynaptic activation via chemical transmission. It has thus been suggested that visual signalling in flies is synchronized with wing flapping at the level of higher brain centres [9] and that visual input primarily activates haltere steering muscles instead of wing steering muscles [53]. The latter studies, however, did not explicitly explain how the signals are able to control the spike phase in wing steering muscles at the cellular level. Alternatively, sensory processing could occur directly within the motor system, which would require sufficient computational power of MN.b1 [54].

Our goal was to develop a numeric simulation that mimics vision-guided flight control of the blowfly *Calliphora* based on dendritic integration processes on the level of a single motoneuron. We used a Hodgkin–Huxley neuron model to simulate the dynamic output of MN.b1 that receives 100 Hz wing stroke-synchronous trains of action potentials from mechanoreceptors and graded membrane potentials from descending visual IN. The simulation shows that subtle shifts in interneuron membrane potential significantly modulate the spiking phase, similar to those values experimentally observed in M.b1 during optomotor stimulation. Moreover, variation of simulation parameters reproduces visual gating and typical activation patterns observed in other steering muscles and neck motoneurons of flies. Based on these results, we discuss strategies for multimodal muscle control in the flight apparatus of flies.

## 2. Model formulation

### 2.1. Hodgkin–Huxley motoneuron model

The core of the simulation is a Hodgkin–Huxley type neuron model [55] of the M.b1 motoneuron (MN.b1) in *Calliphora* that changes its membrane potential d*U*_MN_/d*t* with time *t*, depending on transmembrane currents, and is written as,2.1
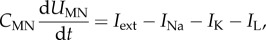
with *C*_MN_ the membrane capacitance per unit area, *I*_ext_ the sum of all electrical currents via electrical synapses between motoneuron and sensory axons, *I*_Na_ the sodium current, *I*_K_ the potassium current and *I*_L_ the neuron's passive leak current (figure 1*d*). The leak current is *I*_L_ = *g*_L_(*U*_MN_ − *E*_L_) with *g*_L_ the constant leak conductance and *E*_L_ the reversal potential of the leak current. Sodium and potassium currents are defined as 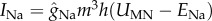 and 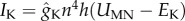, with 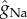 and 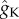 the maximum ion channel conductances, and *E*_Na_ and *E*_K_ the reversal potentials, respectively. The parameters *m*, *n* and *h* are dynamic variables that describe the voltage-dependent activation of sodium and potassium channels, and the sodium channel deactivation after spike initiation, respectively.

The external current *I*_ext_ was computed from synaptic conductance and the difference in membrane potentials between MN.b1 and its sensory inputs, i.e. the potentials of visual IN and campaniform sensilla action potentials from the ipsilateral wing and haltere. The simulated wing stroke period was 10 ms, which is similar to stimulation frequencies used in previous experimental studies on steering muscles [25,29]. The haltere nerve contains approximately 400 [16] afferent axons of which approximately 110 sensilla project onto MN.b1 [29,56]. By contrast, the wing nerve contains about approximately 900 fibres from innervated bristles and sensilla [8]. In this case, the number of axons that project onto MN.b1 is unknown. Owing to similar evolutionary development of wing and haltere campaniforms [24], we simulated 110 sensilla of wing and haltere nerves each (total 220 sensilla).

We excluded modelling *A-type* ion currents that are linked to delayed spike initiation and low firing frequencies in *Drosophila* motoneurons [57]. Their contribution to motoneuron depolarization behaviour is cell-specific [58] and no data are published for MN.b1. Moreover, simulations of slowly firing (approx. 40 Hz) indirect flight muscle motoneurons suggest that two conventional Hodgkin–Huxley type currents (Na^+^, K^+^) are sufficient to reproduce all neural activation patterns [57]. *A-type* currents might thus impair spike behaviour at flight initiation but are not required for establishing spike patterns that are present during continuous flight.

We modelled a single neural compartment with axonal properties because both wing and haltere afferents establish axo-axonal synapses [56] on MN.b1. Visual descending neurons, such as DNDC3-6, likely contact the MN.b1 neuropil via large dorsal dendrites [13]. The latter transmission is modelled by synaptic conductance for visual signalling (*g*_INs_) that determines graded current inputs to the MN.b1 axon. The term *g*_INs_ incorporates both the conductance of the interneuron synapses and the conductance of the dendrite. Adding more compartments to the model would only split these two processes into two separate steps without having significance for the results of our study.

### 2.2. Simulation of haltere and wing signalling

In each stroke cycle, sensilla feedback typically generates a time series of several phase-locked action potentials (spikes) inside the haltere nerve, termed spike volley [8,16]. Experiments suggest that volleys result from cells with different response latencies to mechanical stimuli onto the haltere's sensory fields or from response jitter (figure 1*d*) [20]. In blowflies, 35–55 sensilla are activated during each stroke cycle in undisturbed tethered flight. During moderate 360° s^−1^ yaw turning, the number of active sensilla increases by at least 30% [16]. This suggests an elevated number of active sensilla of more than 45–72 during saccadic turning at yaw velocities of approximately 1600° s^−1^ [59]. Volley duration lasts up to 25% of the stroke cycle in blowflies [16] and 17% in crane flies [19]. The latter study suggests that in crane flies, each campanifom sensillum fires at its own unique phase, covering phases from approximately 7% to approximately 75% stroke cycle [20]. The above estimates must be viewed with caution though, either because of uncertainties in the data traces [16] or because the halteres were externally moved by an electric motor with small flapping amplitude [19,20]. We thus simulated and tested multiple numbers of spiking sensilla and also a broad range of volley durations at the simulated stroke frequency of 100 Hz.

**Figure 2.**
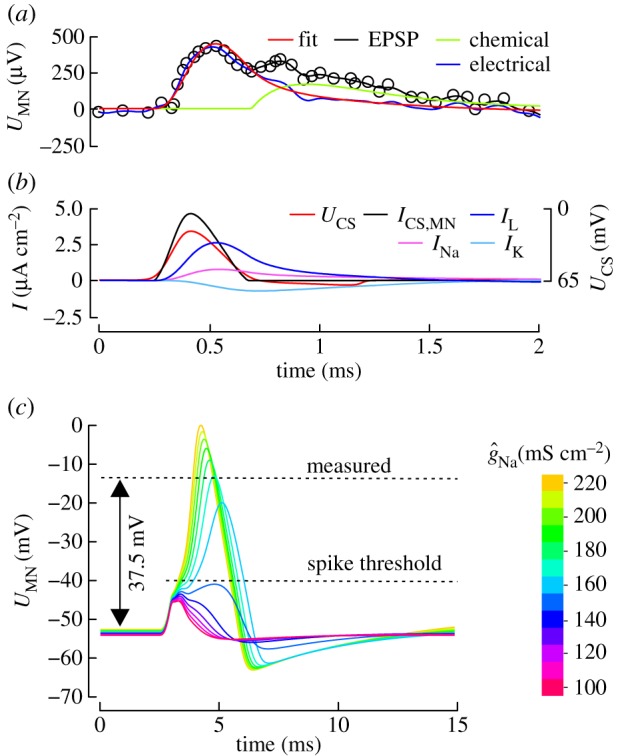
Parameter estimation. (*a*) Mechanical stimulation of a single haltere sensillum in *Calliphora* results in an approximately 0.5 mV postsynaptic potential (black). Data are replotted from [29]. The response consists of an electrical (blue) and a chemical component (green). Parameter values *g*_H_, *g*_L_ and *E*_L_ were estimated from a numerical fit (red) to the electrical component only. (*b*) Motoneuron currents in response to a spike of a single sensillum. *I*_CS_, electrical current through the electrical sensilla-MN.b1 synapse; *I*_L_, *I*_Na_ and *I*_K,_ are leak, sodium and potassium currents through MN.b1 membrane, respectively; *U*_CS_, sensillum membrane potential. (*c*) Simulated membrane potential of MN.b1 (*U*_MN_) in response to a narrow volley of 50 sensilla spikes from the haltere (*σ*= 0.2 ms, cf. figure 1). MN.b1 spike amplitude increases with increasing maximum sodium conductance, 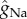. At 165 mS cm^−2^, MN.b1 spike amplitude is approximately equal to the experimentally measured response in *Calliphora* [29]. MN.b1 spike threshold is −40 mV.

As the response latency of MN.b1 to wing nerve stimulation (approx. 1.7 ms) is somewhat larger than to haltere nerve stimulation (approx. 0.9 ms) [25,29], sensilla volleys from the wings should reach the modelled neuron with a temporal delay (*τ*) with respect to the haltere signal. Parameter fitting suggests that the maximum phase response in our model cell occurs at *τ* = 0.625 ms, which is close to approximately 0.8 ms measured by electrical nerve stimulation [25,29]. For simplicity, we did not model sensilla spikes using the Hodgkin–Huxley equations but digitized their waveform from intracellular sensilla recordings in *Tipula* [19]. We rescaled the waveform in time to complement the measured duration of extracellular potentials in the haltere nerve of *Calliphora,* assumed a conventional membrane resting potential of −65 mV, and used a spike amplitude of 50 mV according to previous measurements [20] (figure 2).

### 2.3. Synapse properties

Sensilla spike volleys from halteres and wings produce electrical currents through chemical synapses and gap junctions of steering muscle motoneurons. At natural wing stroke frequencies, the chemical component fades owing to synaptic fatigue, whereas the electrical component quickly stabilizes within several stroke cycles after stimulus start [25]. A data fit to the chemical transmission component [25] suggests that chemical transmission decreases below 10% of its initial value within approximately 130 wing strokes, which equals to only approximately 1.3 s flight time (exponential fit, *y* = a^b^^*x^, *a* = 0.706, *b* = −0.018, *R*^2^ = 0.86). As flies continuously steer for many minutes, a 1.3 s transient is of little functional relevance. Thus, while chemical synapses might play a role during flight initiation, chemical transmission seems to be depleted during steady flight. We therefore exclusively implemented electrical synapses for wing and haltere sensilla in the model.

Currents through electrical synapses depend on the difference between the membrane potentials of sensory axons and MN.b1, and synaptic conductance. A previous study showed that in *Calliphora*, conductance (*g*) is constant over the entire physiological range of spike frequencies [25]. This finding simplifies modelling because in this case, instantaneous currents only depend on the temporal volley structure and the sensory neuron's membrane potential. We determined conductance for halteres (*g*_H_) and wings (*g*_W_) by a multi-dimensional fitting procedure, in which we determined multiple model parameters according to electrophysiological recordings of fly motoneurons (see the next section, figure 2*a,b*). Assuming the same signal structure in all sensilla and same conductance for all synapses, haltere (*I*_H_) and wing (*I*_W_) nerve-induced total currents through an electrical synapse can be written as,2.2
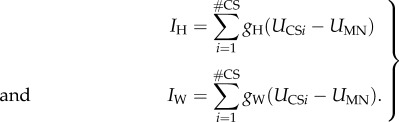
By contrast, visual signalling is transmitted through synapses of up to 50 pairs of descending IN [13]. MN.b1 perceives non-spiking, gradually changing input from at least one of these IN [13]. As dye-coupling studies found electrical synapses but no direct evidence for the existence of chemical synapses [12,13], visual input was exclusively modelled via gap junctions and thus similar to proprioceptive input. Even assuming that chemical transmission exists, the model results would not change because of slow input dynamics and rectification of chemical transmission (see the next section). We considered the combined effect of *k* active visual IN, driving a total current *I*_IN_ into the MN.b1,2.3
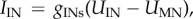
with 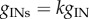. In all experiments, *g*_INs_ was 1.0 mS cm^−2^ (see also electronic supplemental material).

All electrical synapses were modelled as rectifying junctions. Rectification is often seen between different classes of neurons such as visual IN and motoneurons, resulting in an unidirectional flow of information [60]. In *Drosophila*, rectifying synapses were found between the lateral giant fibre and the motor giant neuron, and between several other neurons [61,62]. Direct evidence for rectification also exists for MN.b1 [29]. The latter study showed that there is no response in the haltere nerve when MN.b1 generates spikes, whereas spiking of haltere sensilla results in excitatory postsynaptic potentials (EPSPs) of MN.b1. Thus, the total current through all electrical synapses is,2.4
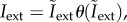
in which *θ* is the Heaviside step function,2.5
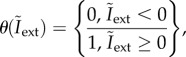


with,2.6
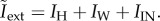


### 2.4. Parameter estimation

Postsynaptic electrical response of MN.b1 to synaptic input depends on two factors. First, the synaptic conductance of the synapse that determines the transmission factor, and, second the leak current dynamics (*g*_L_, *E*_L_) that dominates the subthreshold response towards small currents. We simultaneously determined the parameters *g*_L_, *E*_L_ and *g*_H_ using our Hodgkin–Huxley model, so that the model output matches the EPSP recorded in MN.b1 of *Calliphora* in response to a spike in a single sensillum [29] (figure 2*a*,*b*). The fitting procedure was a multi-dimensional, unconstrained, nonlinear minimization approach on the EPSP's electrical component.

Owing to the initial coexistence of electrical and chemical synaptic transmission, the measured EPSP in MN.b1 consists of an approximately 500 µV fast peak and a second, slowly decaying calcium-peak from the chemical synapse (figure 2*a*). To neglect chemical transmission, we approximated the response of the chemical synapse by a peak-fitting algorithm (peakfit.m, written by Thomas C. O'Haver, exponential pulse waveform) and subtracted the computed fit values (chemical component, green, figure 2*a*) from the measured EPSP (black, figure 2*a*) before application of the Hodgkin–Huxley model fit. The difference is shown in blue and the fit to this difference using the Hodgkin–Huxley model in red (figure 2*a*). To avoid risks associated with overfitting, we tested a set of 100 randomly distributed initial parameter conditions for *g*_L_, *E*_L_ and *g*_H_ (see electronic supplemental material for detailed results, figure S1). At minimum mean error between model and measured data, the fitting procedure yields *g*_H_ = 0.16 mS cm^−2^, *g*_L_ = 5.84 mS cm^−2,^ and *E*_L_ = −52.26 mV. As the electrical response of MN.b1 following wing nerve stimulation is approximately half the haltere nerve stimulation [25], we defined *g*_W_ = 0.08 mS cm^−2^.

In contrast with EPSP, MN.b1 spike amplitude mainly depends on the instantaneous ratio between leak and sodium currents. At low sodium–leak current ratio, the postsynaptic potentials fail to reach the sodium trigger threshold or the elicited spikes tend to undershoot [63–65]. As instantaneous sodium current depends on maximum sodium conductance 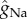, we determined 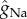 from a comparison between simulated and experimentally recorded spikes in MN.b1. In *Calliphora* and *Drosophila*, MN.b1 spikes reach only small approximately 30–45 mV amplitudes with respect to resting potential [29,30]. Figure 2*c* shows how the simulated spike amplitude increases with increasing 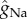. We found that the model output broadly matches the measured mean spike potential of approximately 37.5 mV at 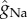 = 165 mS cm^−2^. The remaining model parameters *m*, *n* and *h* were approximated by original Hodgkin–Huxley values. The ratio *g*_L_/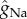 of leak to sodium conductance is 0.035 and similar to the value used in a previous publication on neuron dynamics in *Drosophila* (0.036) [25]. The absolute and relative refractory period of the model neuron are approximately 1.5 ms and approximately 15.5 ms, respectively, and similar to a Hodgkin–Huxley model with standard parameters [66].

The simulation was implemented in Matlab (The MathWorks, USA) partly using a previously published code based on explicit Euler formalism [67]. The adapted code is available on request. To improve accuracy, we modified the code using an explicit second-order Runge–Kutta integration formalism (Heun's method) with an integration time step Δ*t* = 0.01 ms. We validated the model for numerical stability by varying Δ*t* between 0.02 and 0.005 ms.

## 3. Results

### 3.1. Three distinct motoneuron behaviours

**Figure 3.**
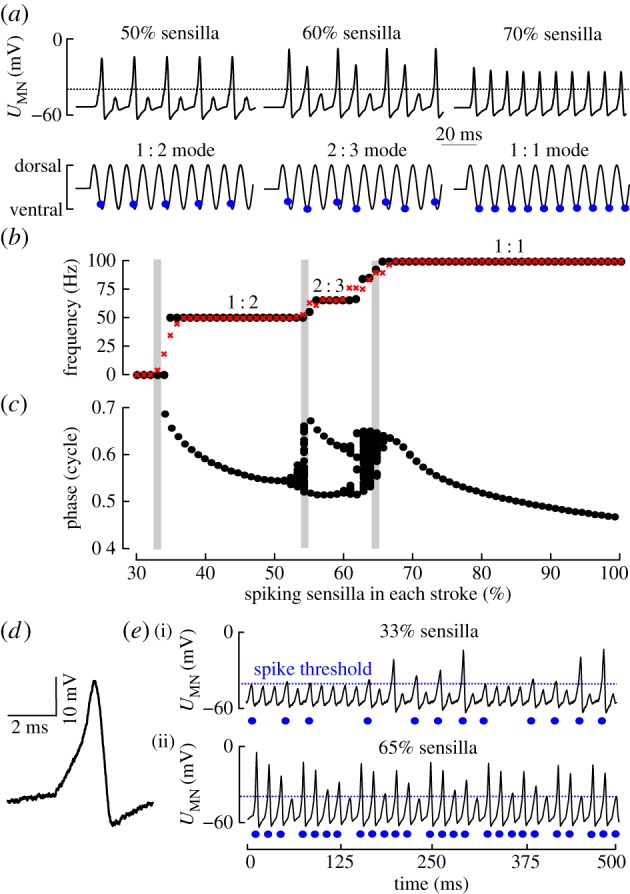
Proprioceptive input determines motoneuron behaviour. (*a*) MN.b1 spiking (upper traces) in response to 100 Hz spike volleys of 55 (left), 66 (middle) and 77 (right) sensilla from wing and haltere, each corresponding to 50, 60 and 70% of total 220 sensilla, respectively. Mean phase of haltere and wing volley is 25 and 31.25% stroke cycle, respectively (*σ* = 1.0 ms, *τ* = 0.625 ms, *U*_IN_ = −35 mV) (*a–d*). Note that synaptic conductance of wing axons is only half of the halteres. Lower traces show wing position and phase of MN.b1 spike (blue). (*b*) MN.b1 firing frequency without (black) and with membrane noise (red, 0.1 mV) increases with increasing number of spiking sensilla. Plotted ratios indicate number of MN.b1 spikes per number of stroke cycles. (*c*) MN.b1 spike phase without membrane noise. At transients between locking intervals (grey), spike generation occurs in an irregular manner. (*d*) Simulated MN.b1 spike with added membrane noise (*σ*_Noise_ = 0.25 mV). (*e*) Upper trace: fluctuations of MN.b1 potential owing to membrane noise lead to intermittent MN.b1 spiking (blue dots) at 33% sensilla activity. Lower trace: irregular firing pattern at 1 : 1 locking (65% active sensilla, without membrane noise).

During flight, MN.b1 and other steering muscle motoneurons perceive periodic input from wing and haltere campaniform sensilla in every wing stroke [8]. Up to now, there are no data available on the number of spiking sensilla in freely manoeuvring flies. We thus simulated different input strength from a minimum of one haltere and one wing sensillum to a maximum of 110 sensilla each. We found three distinct behaviours of the model cell that are consistent with previous experimental and numerical studies on neurons subject to periodic input [68]. These behaviours are *quiescence*, *frequency-locking* and *irregular firing* (figure 3). In this section, we explain how these behaviours depend on the number of active sensilla. The results from a systematic parameter mapping procedure are presented in the following section.

Figure 3*b* shows that no motoneuron spikes are generated if the number of spiking sensilla is less than 35% of total 220 sensilla (quiescence behaviour). The quiescence behaviour persists when visual input varies between −40 and −30 mV. Above a lower stimulus threshold of 35% spiking sensilla, the input entrains the model cell to fire. At an input between 35 and 55% spiking sensilla, the model regularly fires a single spike in every second wing stroke (1 : 2 locking behaviour, figure 3*a,b*). At an input between 57 and 63%, the motoneuron generates two spikes every three wing strokes (2 : 3 locking behaviour), and input of more than 67% sensilla entrains spiking in every wing stroke (1 : 1 locking behaviour) and thus at wing stroke frequency. A value of 67% compares to 148 sensilla, i.e. 74 sensilla from haltere and wing each. At 1 : 2 and 1 : 1 locking behaviours, all motoneuron spikes are generated at one stroke cycle phase, depending on input strength. At 2 : 3 locking behaviour, by contrast, spike phase alternates (figure 3*c*). In this case, every second spike is phase-delayed owing to an insufficient recovery time of the model ion channels.

**Figure 4.**
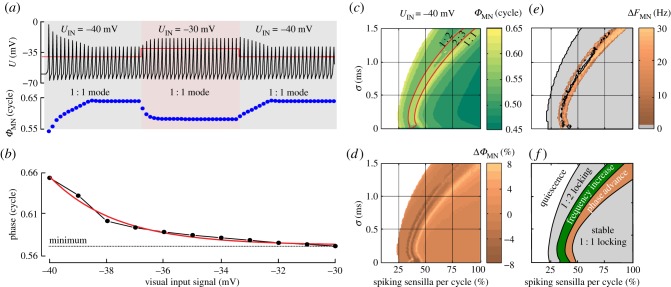
Visual control of MN.b1 spike phase. (*a*) MN.b1 membrane potential (*Φ*_MN_, upper trace) and corresponing spike phase (*Φ*_MN_, lower traces) at 1 : 1 locking. Stepwise 10 mV depolarization of visual input (*U*_IN_, red) leads to an advance in spike phase. Spike volley width is *σ* = 0.2 ms and number of spiking sensilla 42%, which corresponds to 46 sensilla from haltere and wing each (220 total). (*b*) Steady-state MN.b1 firing phase monotonically decreases with increasing membrane potential of visual IN (parameters are equal to *a*). Curve fit function (red) is *y* = 0.574 × e^−*x*/2.34^ with *R*^2^ = 0.99. (*c*) Phase map for variations in sensilla signalling at *U*_IN_ = −40 mV (*τ* = 0.625 ms). Red lines show borders of locking ratios. (*d*) Relative changes in MN.b1 spike phase and (*e*) frequency (*F*_MN_) resulting from interneuron depolarization (*U*_IN_, −40 to −30 mV). (*f*) Schematics of MN.b1 activation modes.

In figure 4*c*, we summarize sensilla-induced locking mode and phase shifting behaviours. This figure shows how an increasing number of spiking sensilla shifts spiking modes and within each spiking mode also spiking phase. During 1 : 2 locking, phase decreases (advances) with an increasing number of spiking sensilla until the system switches to 2 : 3 locking. Within 2 : 3 locking, there is little phase effect. Switching to 1 : 1 locking, phase is maximum delayed but decreases (advances) if the number of spiking sensilla further increases.

At the transients between the three locking intervals, spike generation occurs in an irregular manner, producing fluctuating spike frequency (figure 3*e*, lower trace) and phase shift (grey, figure 3*b*,*c*). Spike amplitude varies and spike phases are distributed over an extended interval of approximately 12% stroke cycle. This non-periodic (chaotic) MN.b1 firing in response to periodic input results from the intrinsic nonlinearity of the Hodgkin–Huxley equations and is independent of any membrane noise [69–71].

Neurons typically face electrical membrane noise resulting from various sources that may alter excitability and thus the outcome of our numerical simulation [67]. Experimental data suggest that MN.b1 membrane potential fluctuates with magnitudes up to 0.25 mV [29,30]. To evaluate phase stability during mode-locking under more natural conditions, we added random membrane potentials (Gaussian noise) to the model cell (figure 3*d*). Note that voltage noise differs from current and conductance noise and directly acts on the membrane potential. The added fluctuating potentials are normally distributed around zero with standard deviations (*σ*_Noise_) of 0.1 and 0.25 mV. The latter values represent the lower and upper boundaries in noise that have been previously measured in flies [29,30]. We found that voltage noise has only a limited effect on phase stability during 1 : 1 locking. Standard deviation of spiking phase was approximately 0.7% stroke cycle at *σ*_Noise_ = 0.1 mV and approximately 1.5% stroke cycle at *σ*_Noise_ = 0.25 mV. This robustness against membrane noise supports the assumption that, besides spike frequency, the spike phase is a convenient measure to control steering muscle power in flies [1]. However, noise smears out the sharp transients between spiking modes. Figure 3*e* shows that at subthreshold input of 33% spiking sensilla (upper trace), the membrane fluctuations are sufficient in magnitude to elicit MN.b1 spikes, leading to stochastic spiking with random interspike intervals.

### 3.2. Visual signalling determines motoneuron spike phase

During visual manoeuvring flight, MN.b1 perceives changing electrical currents resulting from graded changes in membrane potential of visual IN. To test the impact on flight control, we added stepwise and oscillatory changes in interneuron potential to our model cell and scored MN.b1 spike phase.

Figure 4*a* shows that at 1 : 1 locking, stepwise depolarization of *U*_IN_ (ON response) from −40 to −30 mV leads to an approximately 9 mV increase in spike amplitude and an advance in spike phase of approximately 10% stroke cycle within three stroke cycles after the transition. The steady-state response at 1 : 1 locking shows that with increasing interneuron membrane potential, MN.b1 spike phase decreases (advances) monotonically from approximately 65% to approximately 57% stroke cycle (figure 4*b*). Repolarization (OFF response) from −30 to −40 mV shows longer transients, scattered around eight stroke cycles (figure 4*a*). Similar to a low-pass filter, ON- and OFF-response times alter phase shift amplitude and timing between visual input and motoneuron phase during oscillatory input. The magnitude of both effects increases with increasing input frequency. At 10 mV peak-to-peak sinusoidal visual stimulation (−30 to −40 mV), stimulus frequencies of up to approximately 2 Hz attenuate phase modulation only little, i.e. peak phase modulation amounts to approximately 95% of the steady-state response. At elevated frequencies, MN.b1 phase modulation progressively collapses and maximum phase shifts are only approximately 75% of the steady-state response at 3 Hz, 63% at 4 Hz, 48% at 8 Hz and 40% at 16 Hz stimulus frequency (data not shown). Noteworthy, a similar stimulus frequency-dependent alteration has also been observed in the wing kinematics of tethered flying *Drosophila*, responding to oscillating visual stimuli displayed in a flight simulator [72].

We further investigated how visual motor control depends on the temporal structure of the mechanosensory input. This analysis included two-dimensional parameter mapping of volley width and number of spiking sensilla. Figure 4*c* shows the parameter combinations that produce the dominant model behaviours: quiescence, 1 : 2, 2 : 3 and 1 : 1 locking at constant visual input (*U*_IN_ = −40 mV). Figure 4*d* shows that a 10 mV depolarization of visual IN (*U*_IN_ = −30 mV) relatively shifts MN.b1 phase by up to approximately 8% stroke cycle. This value, however, requires specific combinations between number of spiking sensilla and volley widths. Our model predicts that phase advances during 1 : 1 locking are limited to a small band of input parameters near the transition to 2 : 3 locking (light brown, figure 4*d*). Within this transition, the firing behaviour of MN.b1 can vary between 1 : 1 and 2 : 3 locking due to changing visual signalling, altering both spike phase and frequency (figure 4*d,e*). Experimental studies on tethered flies report similar results on wing stroke frequency fluctuations in flight [31,73]. Our simulation suggests that this variance in firing might result from an insufficient number of spiking sensilla required for reliably firing spikes in 1 : 1 mode at tethered flight condition.

### 3.3. Visual gating

**Figure 5.**
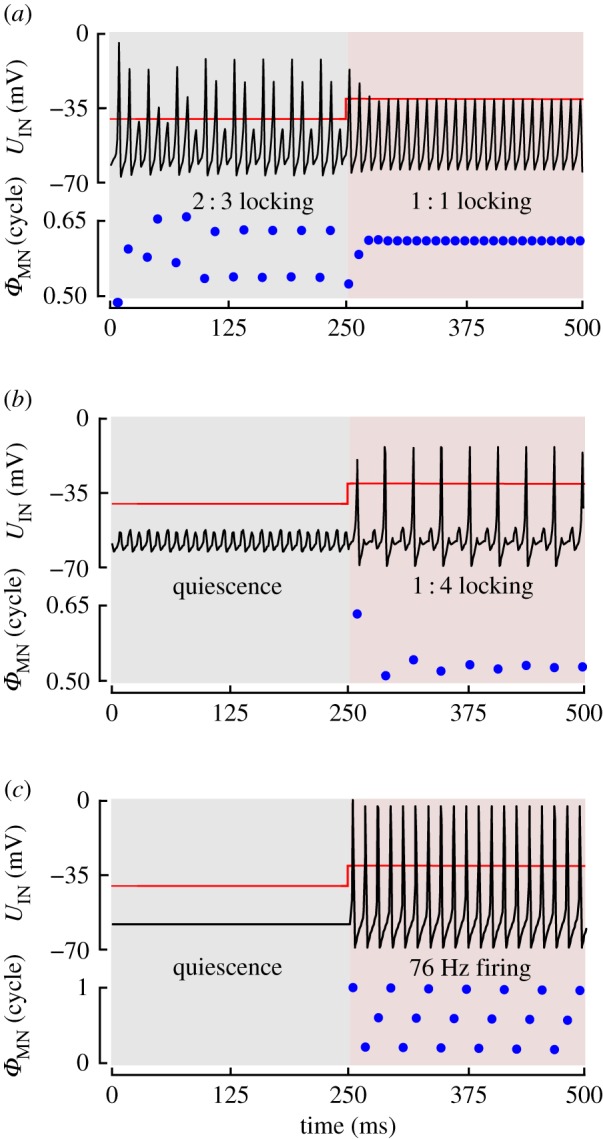
Mode switching and visual gating. (*a*) Example of mode switching at physiological parameters of the model cell. At *σ* = 0.72 ms and 42% spiking sensilla, a 10 mV depolarization of visual input shifts 2 : 3 motoneuron firing mode to a 1 : 1 firing mode with stable phase. (*b,c*) A model cell with increased electrical excitability allows visual gating of phase-locked (15% spiking sensilla, *g*_L_ = 3.6 mS cm^−2^, *ĝ*_Na_ = 165 mS cm^−2^, *σ* = 0.5 ms) in *b* and non-locked motoneuron spiking (0% sensilla, *g*_L_ = 5.86 mS cm^−2^, *ĝ*_Na_ = 240 mS cm^−2^, *σ* = 0.5 ms) in *c*.

With parameter settings that approximate the physiology of an M.b1 motoneuron, our model may not reproduce visual gating of motoneuron spiking. Visual gating is typical for steering muscle motoneurons of axillary M.I1, M.III1 and basalare M.b2 [32] including neck muscle motoneurons [74]. Once gated in flight, spikes are phase-locked with respect to the stoke cycle [74]. We found that visual gating only occurs in the model, if we increase neuronal excitability of the simulated membrane by increasing maximum conductance of sodium channels 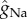 and/or decreasing leaky conductance *g*_L_. Notably, these changes are not covered by experimental data but highlight that relatively small modifications in model parameters are sufficient to produce gating and other spiking behaviours. The latter is important because of the uncertainties in experimental data and our little knowledge on properties of other flight motoneurons than MN.b1. Spike gating is similar to a shift in spiking mode at physiological conditions (MN.b1 parameter values), as shown for the response to a 10 mV change in visual input in figure 5*a*. In contrast with figure 5*a*, however, visual gating occurs between quiescence and locking behaviours. The following two examples are chosen to highlight this issue.

Figure 5*b* shows that spike gating behaviour occurs when *g*_L_ is reduced from 5.84 to 3.60 mS cm^−2^, while keeping all other Hudgkin–Huxley parameters. At these settings, spiking of few (13–26) sensilla produces small EPSPs below the firing threshold. A depolarization in visual interneuron potential by 10 mV allows spike initiation with ¼ stroke frequency (1 : 4 locking behaviour). This pattern is close to what has been observed in M.b2 during flight in *Drosophila* [74]. Figure 5*c* shows a simulation, in which 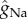 is reinforced from 165 to 240 mS cm^−2^. Under these conditions, the model neuron spikes at low 76 Hz in response to 10 mV depolarization of the visual interneuron even in the absence of proprioceptive feedback and thus without phase-locking.

## 4. Discussion

### 4.1. Model robustness and significance of inputs

We rigorously tested the robustness of our findings using various combinations between *τ*, *σ*, *g*_INs_ and *U*_IN_ (see electronic supplementary material, figure S2). We found that the required number of spiking sensilla for 1 : 1 mode-locking increases with increasing *σ*, *τ* and *g*_INs_, and decreases with increasing (depolarizing) *U*_IN_. We also found that at a wide physiological range, multiple combinations of model parameters are able to produce MN.b1 1 : 1 phase-locked firing behaviour. This phenomenon is known from other modelling studies and reflects findings that neural circuits of individuals may robustly yield the same output, even if there is a considerable variance in neuronal properties [75]. Variance is also present in genetically identical flies, such as in the number and properties of synapses, ion channels and receptors [76].

Vision-induced phase shifting, by contrast, appears to be limited to rather specific combinations of sensilla spike number and the spikes' temporal distribution (figure 4*d*). Thus, considering the variability of biological systems, the question arises how MN.b1 maintains its finely tuned properties over the entire lifetime of the fly because numerous studies show that the physiology of a fly changes as the animal grows older [77–79]. One possible explanation is homeostatic control of MN.b1. In particular, the activity-dependent regulation of ion channel densities that determine signalling properties. Homeostatic control may stabilize neural function over time by constraining neural plasticity [80]. For example, it has been shown that homeostatic control of signal transmission at the *Drosophila* neuromuscular junction (NMJ) operates rapidly on a timescale of seconds [81] and with millivolt precision [82]. Homeostatic control might also explain how flies that were allowed to rest for 1 h after partial ablation of mechanosensory inputs are able to perform visual manoeuvring under tethered conditions [28].

In contrast with a standard Hodgkin–Huxley neuron, the membrane of our model neuron is less electrically excitable. This is largely due to an approximately 20 times higher leak conductance (*g*_L_ = 5.84 mS cm^−2^ versus *g*_L_ = 0.3 mS cm^−2^, MN.b1 versus standard neuron), while maximum sodium conductance is approximately similar in both types of neurons (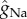
= 165 mS cm^−2^ versus 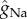 = 120 mS cm^−2^). Nevertheless, major properties of our MN.b1 model cell such as sodium conductance and the ratio between leak and sodium conductance (*g*_L_/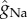
= 0.0354) are almost identical to experimental values obtained from motoneurons of indirect flight muscles in *Drosophila*, i.e. 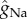
= 156 mS cm^−2^ and *g*_L_/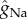
= 0.036 [57]. The reduced electrical excitability of MN.b1 model membrane hinders our model cell to spike in response to vision without proprioceptive input. This means that MN.b1 may not generate spikes in a resting fly, even though the visual system provides strong excitatory input from a moving environment (cf. §3.3). Notably, this suppression of motoneuron activity was also found in electrophysiological measurements in blowflies [53], in which it was shown that visual stimulation of resting flies does not generate any spikes in wing steering muscles.

### 4.2. Multimodal flight control

Major results of our simulation are consistent with multiple behavioural and electrophysiological findings in flies. In particular, the simulation suggests a mechanistic explanation for the various forms of spike patterns and phase-locking behaviours as measured in flight steering muscles. We limited our analysis to steering muscles because asynchronous power muscles in flies morphologically and functionally differ from steering muscles [8,83].

**Figure 6.**
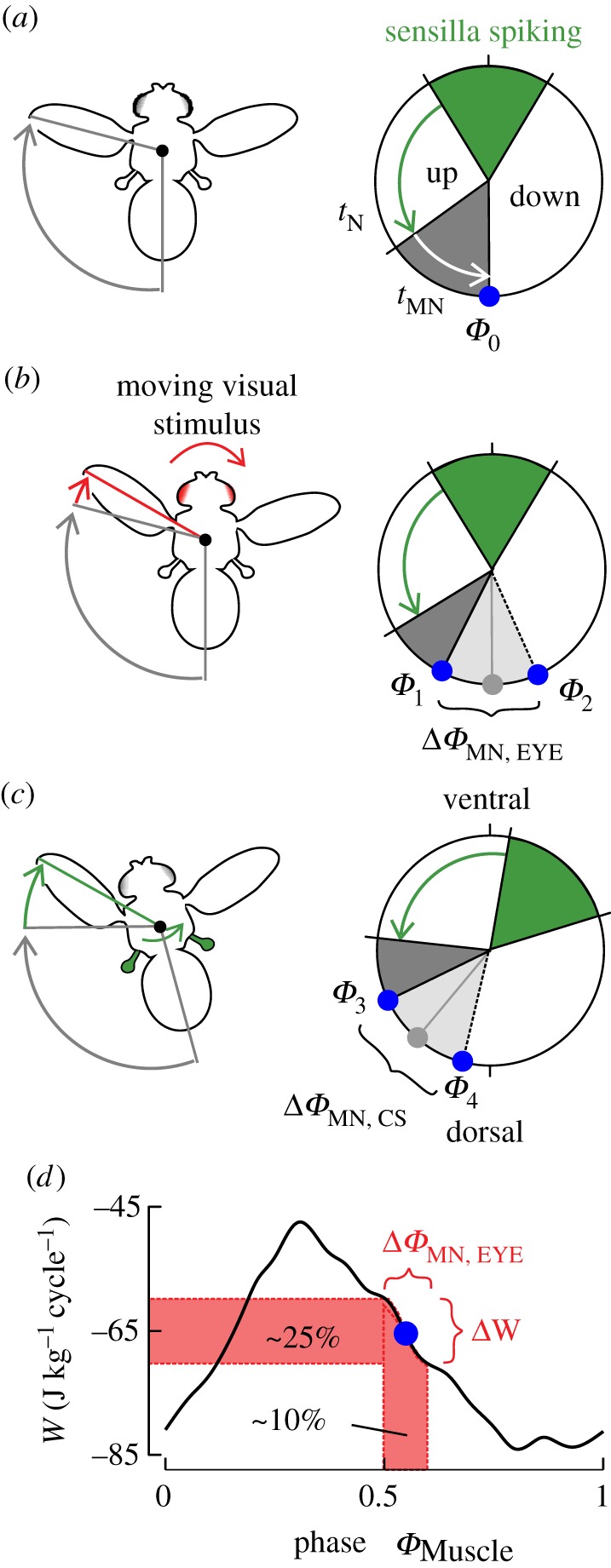
Principles of multimodal flight control. (*a*) During unperturbed straight flight, sensilla spiking near the ventral stroke reversal (0% stroke cycle) initiates one spike in MN.b1 at phase *Φ*_0_. Delays are due to spike propagation (*t*_N_) via the haltere nerve and time for motoneuron depolarization (*t*_MN_). (*b*) Optic flow on the retina of the compound eye transiently increases (decreases) interneuron membrane potential, which decreases (increases) *t*_MN_ and thus advances (delays) spike phase (Δ*Φ*_MN,EYE_). (*c*) Body rotations generate Coriolis forces that transiently temporally advance sensilla spiking and/or increase the number of active sensilla. These changes cause an advance of MN.b1 spike phase (Δ*Φ*_MN,CS_), owing to a decrease in *t*_MN_. (*d*) The muscle work-phase relationship of M.b1 in *Calliphora* indicates that an approximately 10% shift in spike phase leads to an approximately 25% change in muscle work (*W*) during oscillatory length changes of the muscle (replotted from [1]).

Our data show how the number and temporal distribution of spiking sensilla and visual input alter the output of MN.b1 (figures 3–5). Besides vision-guided wing control, our modelling also provides a possible explanation for the function of the fly's ‘gyroscopic autostabilizer’ (figure 6*c*) [18,84]. The autostabilizer describes a feedback control-loop in flies, with which the haltere output controls wing motion by gyroscopic sensing [18,84]. This feedback-loop is thought to stabilize the fly's body without visual input [18,84]. Body rotation, for example, changes Coriolis force on halteres and wings. While halteres are thought to be deflected out-of-plane, wings may undergo torsional deformation during body rotation [18,84]. The resulting cuticular stress on the structures may recruit additional sensilla and likely increases spike volley width. According to our simulation results, rotation-induced changes in sensory input may lead to a change in MN.b1 spike mode, assuming a small number of active sensilla, or a Coriolis force-dependent advance in MN.b1 spike phase at an elevated number of spiking sensilla (1 : 1 locking). Figure 4*d* also suggests that during 1 : 1 locking, an increase in the number of spiking sensilla decreases the impact of the visual system on phase control. In other words: if the halteres get more active, they more and more disable vision-induced phase control. The spike phase, however, still advances with increasing numbers of spiking sensille, as shown in figure 4*c*. This means for example, that during quick yaw turns, haltere output modulates muscle tension, while vision-induced phase changes are broadly suppressed. The latter prediction might explain the experimental finding that mechanical oscillation of the fly's body leads to suppression of vision-induced steering behaviour [54].

As already mentioned in the introduction, flight steering muscles in flies fall into two groups: tonic and phasic muscles [34]. Tonic muscles are typically continuously active in flight such as M.b1, while phasic muscles sporadically generate action potentials [8]. Phasic muscles may be gated by the visual system [32]. The spikes in both types of muscles are phase-coupled to the stroke cycle [8]. Tonic muscles subtly change wing motion as needed for smooth manoeuvring, body stabilization and fine-tuning of body saccades [34,52]. Gated muscles, by contrast, control more elevated changes in wing kinematics (figure 5*a–c*) [49]. Assuming that the properties of motoneurons vary between the various steering muscles and that visually gated motoneurons exhibit elevated membrane excitability (cf. §3.3), our model reproduces both—phase control and gating. Most notably, maximum visually induced phase shift of the MN.b1 model neuron (approx. 9% stroke cycle, figure 4*b*) is consistent with experimentally derived values on M.b1 in *Calliphora* and *Drosophila.* In tethered animals, these values range from 10 to 15% stroke cycle [8,31,32]. A 10% shift in activation phase thereby translates into a 25% change in muscle mechanical output of M.b1 (figure 6*d*) [1,49].

The question of how steering muscles produce different behaviours to similar proprioceptive and visual input is puzzling. A possible explanation is as follows. Our simulation of visual control identified a lower threshold value for *U*_IN_ that is needed to elicit spike gating in MN.b1. In the selected example (figure 5*b*), this value amounts to approximately −34 mV, i.e. approximately 6 mV above resting potential. However, a depolarization of approximately 6 mV is also sufficient to shift MN.b1 phase by approximately three-fourths of its maximum value. We thus hypothesize that if visual IN remain subthreshold owing to weak visual input, the flying animal will smoothly adjust its wing motion only by phase shift in tonic muscles, such as M.b1. At elevated visual stimulation, interneuron depolarization may exceed the threshold for muscle spiking. As a consequence, the fly could recruit visually gated muscles for elevated changes in wing kinematics, such as M.b2 [2]. Under free flight conditions, there might be continuous switching and overlap between these two flight modes, depending on the strength of feedback from the visual environment, e.g. during self-motion. Descending neurons might also perceive input from other sensors such as the antennae and higher-order commands from the central brain. This additional input might explain that visually gated steering muscles do not always spike in the presence of visual stimuli [12,34]. In sum, a comparatively simple change in electrical excitability of the motoneuron membrane might be responsible for the different response behaviours of steering muscles, so that muscles with different properties can be controlled by the same underlying neural circuitry.

## 5. Concluding remarks

For decades, flight control in flies served as a model system for understanding basic mechanisms of neural computation during locomotion. The flight motor system that structures the underlying motor pattern has usually been considered as a ‘black box’ and treated with control-theoretical and descriptive models [21,49,54,85–89]. Although high-level models identify functional features, they can hardly explain how sensory information is processed on a cellular level. Without seeking higher brain function, our model reproduces several experimental findings and generates hypotheses for their underlying neural function. In flies, the computational power of a single motoneuron appears to be adequate for sensory integration, allowing precise phase-dependent changes in wing muscle power. Our simulation further suggests that there is no need to synchronize visual signalling with the wing stroke before sensory integration, as previously proposed [9]. As the model can be smoothly adapted to generate the firing pattern observed in other motoneurons, the integration of wing stroke synchronous proprioceptive action potentials and graded visual signalling on the level of motoneurons might be a common principle for motor control in flies, and maybe other flying insects.

## Supplementary Material

Parameters and fitting procedure of the Hodgkin-Huxley neuron model

## Data accessibility

All data needed to evaluate the conclusions in the paper are present in the paper itself and in the electronic supplemental materials or are available from the Dryad Digital Repository: http://dx.doi.org/10.5061/dryad.fp3qb51 [90].
