# Supplementary material for: Sensory processing by motoneurons: a numerical model for low-level flight control in flies
Source: J R Soc Interface. 2018 Aug 29;15(145):20180408. doi: 10.1098/rsif.2018.0408 (PMC6127168; doi:10.1098/rsif.2018.0408)
Supplement: Parameters and fitting procedure of the Hodgkin-Huxley neuron model [file rsif20180408supp1.pdf]

Journal of the Royal Society Interface: Electronic Supplemental Material

Sensory processing by motoneurons – a numerical model for low-level flight control in flies

Jan Bartussek and Fritz-Olaf Lehmann, July 2018

Institute of Biological Sciences, Department of Animal Physiology, University of Rostock, 18059 Rostock, Germany

Email of corresponding author: fritz.lehmann@uni-rostock.de

Fitting Procedure. We estimated several parameter values of our Hodgkin-Huxley neuron model from experimental data. In particular, we derived values from the time course of an excitatory postsynaptic potential (EPSP) in MN.b1 of *Calliphora* in response to a single spike in its sensory axon from the haltere [Fayyazuddin, A. & Dickinson, M.H. 1996. Haltere afferents provide direct, electrotonic input to a steering motor neuron of the blowfly, *Calliphora*. *J. Neurosci.* **16**, 5225-5232]. We obtained values for (1) the leak conductance, g_L, (2) the conductance of the electrical synapse between haltere afference and MN.b1, g_H, and (3) the reversal potential of the MN.b1 leak current, E_L. We used a multidimensional, unconstrained, nonlinear minimization approach (Matlab function fminsearch.m). To avoid local minima, we tested 100 randomly selected initial values for the three parameters using rmsearch.m, written by John D'Errico. The parameter range for these values was 0.01 to 10 mS cm^{-2} for g_H and g_L, and -60 to -40 mV for E_L. Figure S1 shows that approximately 40% of the tested combinations converge to nearly identical values (grey). It is thus likely that the estimated values are not based on a local minimum of the fit error (Fig. S1*d*). More evidence for the magnitude of the parameters comes from experimental results as described in the manuscript.

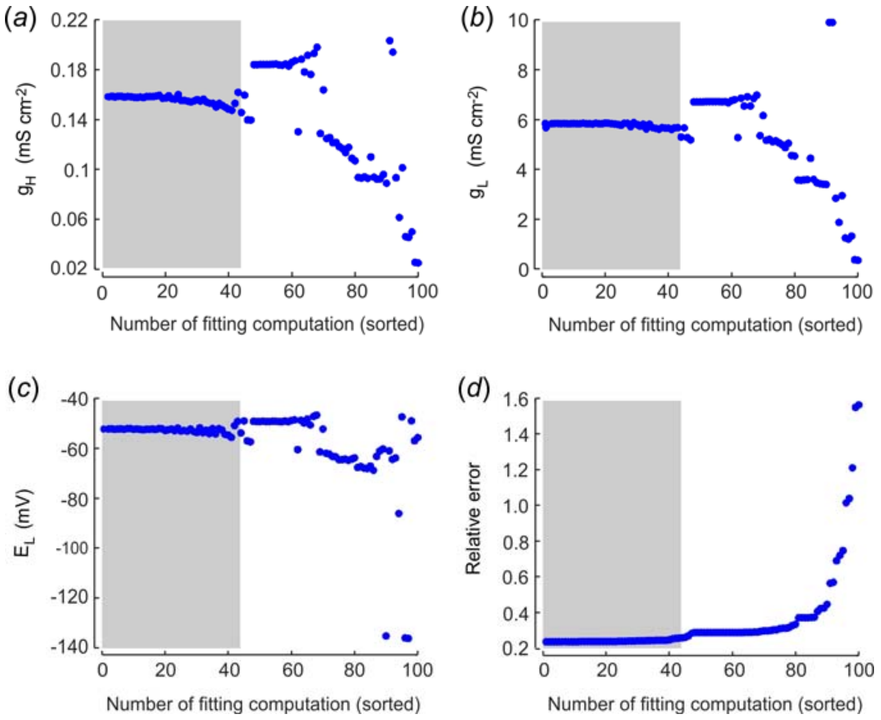

Fig. S1. Results of parameter fitting, sorted by final error value. We tested 100 randomly generated initial values. g_H, leak conductance of MN.b1; E_L, reversal potential of leak current; g_H, conductance of electrical synapse between haltere and MN.b1.

Simulation Parameters for Spike Initiation. To investigate the requirements for 1:1 mode-locking (100 Hz spike frequency), i.e. to obtain one MN.b1 spike in every stroke cycle, we tested the dependencies of the simulation using multiple combinations between the delay of haltere and wing volley (τ), the width of spike volley distribution (σ_{CS}, standard deviation), the conductance of potassium ($\hat{g}_K$) and sodium ($\hat{g}_{Na}$), and the potential of the visual interneurons (U_{IN}). Figure S2 shows how the minimum number of campaniform sensilla from the haltere for 1:1 mode-locking (CS_{min}) changes depending on the model parameters. In figure S1a, constant parameters are U_{IN} = -35 mV, g_{INs} = 1.0 mS cm^{-2}, $\hat{g}_{Na}$ = 165 mS cm^{-2}, and $\hat{g}_K$ = 36 mS cm^{-2}. Data in figure S1b were computed with constants U_{IN} = -35 mV, g_{INs} = 1.0 mS cm^{-2}, σ_{CS} = 0.6 ms, and τ = 1.25 ms. Eventually, in figure S1c the constant values are $\hat{g}_{Na}$ = 165 mS cm^{-2}, $\hat{g}_K$ = 36 mS cm^{-2}, σ_{CS} = 0.6 ms, and τ = 1.25 ms.

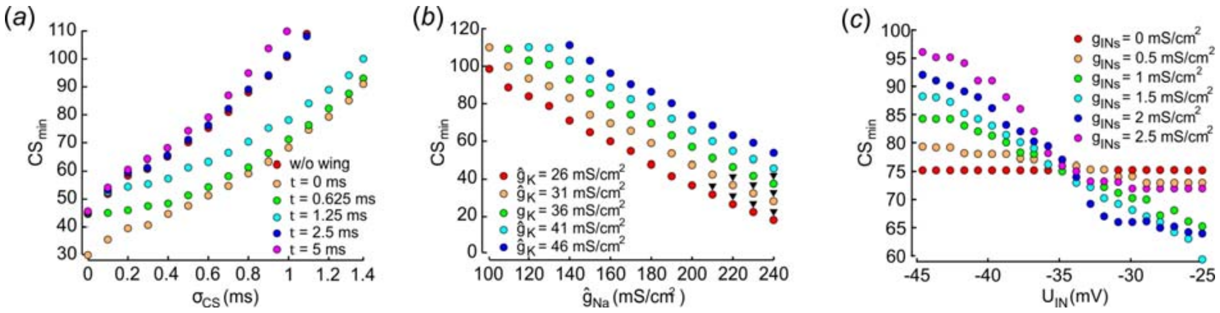

Fig. S2. The minimum number of spiking campaniform sensilla (CS_{min}) from the haltere to elicit 100 Hz spiking in MN.b1 depends on model parameters. (*a*) CS_{min} plotted as a function of spike volley distribution (*x*-axis) and temporal delay between wing and haltere signaling (color). (*b*) CS_{min} plotted as a function of sodium (*x*-axis) and potassium (color) conductances of the modeled neuron. (*c*) CS_{min} plotted as a function of visual signaling (*x*-axis) and conductance of gap junction between visual descending neurons and b1 motoneuron (color).
